# Utilization of Clinical Practice Guideline Interventions in the Conservative Management of Mechanical Neck Pain: A Retrospective Analysis

**DOI:** 10.7759/cureus.34794

**Published:** 2023-02-09

**Authors:** Anthony Baumann, Michelle Youngquist, Deven Curtis, Mingda Chen, Keith D Baldwin

**Affiliations:** 1 Rehabilitation Services, University Hospitals Cleveland Medical Center, Cleveland, USA; 2 Medicine, Northeast Ohio Medical University, Rootstown, USA; 3 Medicine, Case Western Reserve University, Cleveland, USA; 4 Orthopedics, Children's Hospital of Philadelphia, Philadelphia, USA

**Keywords:** cervicalgia, manual therapy, clinical practice guidelines, physical therapy, neck pain

## Abstract

Introduction: Neck pain is a common musculoskeletal condition frequently treated by physical therapists. The American Physical Therapy Association (APTA) published a clinical practice guideline (CPG) in 2008 with a revision in 2017 to improve the diagnosis and treatment of neck pain. One subset of neck pain in the CPG is “Neck Pain with Mobility Deficits,” also called mechanical neck pain. Little data exists on the adherence of physical therapists to the CPG-recommended treatments for neck pain as well as the outcomes associated with the utilization of the CPG. The purpose of this study is to examine both CPG treatment adherence and associated outcomes in patients treated for mechanical neck pain by physical therapists in the outpatient setting.

Methods: Retrospective chart review of patients (n=224) who received physical therapy for neck pain between 2018 and 2022. Data ranges were chosen due to the publication of the CPG revision in 2017. Six interventions for mechanical neck pain from the CPG were examined: thoracic manipulation, cervical mobilization, transcutaneous electrical stimulation (TENS), dry needling, advice to stay active, and scapular resistance exercises. The exclusion criteria were a history of cervical spine surgery. Other data collected included age, sex, characteristics of the evaluating physical therapist, and the number of visits.

Results: For CPG treatment adherence, 4.5% of patients received thoracic manipulation, 47.8% of patients received cervical mobilization, 12.5% of patients received TENS, 22.8% of patients received dry needling, 99.1% of patients received advice to stay active, and 89.3% of patients received scapular resistance exercises. There was no significant improvement in pain, range of motion (ROM), and function based on a number of CPG interventions used during the bout of physical therapy (p=0.17 to p=0.74). Patients who were evaluated by a physical therapist who was an Orthopedic Certified Specialist (OCS) were more likely to receive more interventions recommended by the CPG (p<0.01).

Conclusion:CPG-recommended treatments are used with varying frequency by physical therapists when treating mechanical neck pain. Thoracic manipulation is rarely used while scapular resistance exercises are frequently used. There was no significant improvement in pain, ROM, or function based on the number of CPG-recommended treatments used during the bout of physical therapy.

## Introduction

Neck pain is a common musculoskeletal pathology treated by physical therapists around the world [1,2]. Neck pain is one of the highest complaints in terms of years lived with a disability, increasing the relevancy of proper management by physical therapists [1,3]. The overall prevalence of neck pain ranges from 10% to 20%, and the incidence of new neck pain ranging from 10% to 50% [1,3]. In an effort to improve patient care, the American Physical Therapy Association (APTA) in 2017 recently updated the 2008 Neck Pain Clinical Practice Guideline (CPG) to help physical therapists diagnose and treat different subsets of neck pain [1,2]. One subset of neck pain listed in the CPG is “Neck Pain with Mobility Deficits”, otherwise called mechanical neck pain [1].

Common signs and symptoms of mechanical neck pain as listed in the CPG include central and/or unilateral neck pain, limited cervical range of motion (ROM), neck pain that is reproduced at end ranges of ROM, and restricted cervical and thoracic segmental mobility [1]. This diagnosis is in contrast to other subsets of neck pain in the CPG, such as "Neck Pain with Radiating Pain" and "Neck Pain with Movement Coordination Impairments" [1]. While many different non-surgical interventions have been presented in the literature for the management of neck pain (such as manual therapy, exercise, patient education, and physical agents like ice and heat), the Neck Pain CPG strives to provide evidence-based recommendations regarding interventions for neck pain to allow clinicians to treat their patients in a more effective manner [1]. However, little is known about the actual utilization rate or outcomes associated with using the CPG in clinic practice.

One recent study found that utilization of neck and low back CPGs was about 70%; however, utilization was not associated with final pain or disability in the study cohort [4]. A great deal of interest in the literature is focused on utilization of other clinical practice guidelines [5-8]. Much of the current data focuses on adherence to other CPGs, such as for low back pain or concussion [9,10]. However, many of the studies that examine adherence to CPG recommendations are surveys or clinician interviews, which may cloud actual CPG adherence due to recall bias and unintentional positive view of one’s own treatment plans [6,9,10]. The purpose of the current study is to examine the adherence of physical therapists to recommended treatments within the CPG as well as outcomes associated with CPG utilization in patients with mechanical neck pain.

## Materials and methods

The current study is a retrospective chart review of 224 patients from multiple outpatient clinics within a single hospital system from 2018 to 2022. The current study was approved by the University Hospitals Institutional Review Board (IRB) under IRB approval #20220542. Patient charts were gathered by the University Hospitals Clinical Research Center based on having a diagnosis of neck pain (ICD-10 code M54.2) as well as a physical therapy evaluation procedure code (97161, 97162, or 97163). Inclusion criteria include receiving physical therapy for neck pain, being older than 18 years old, attending at minimum two sessions of physical therapy (one evaluation session and at least one treatment session), and fitting best into the CPG category of “Neck Pain with Mobility Deficits” based on information in the patient chart. Exclusion criteria were a history of cervical spine surgery and fitting the CPG categories of “Neck Pain with Radiating Pain” or “Neck Pain with Movement Coordination Impairments.” From a total of 1500 patient charts, 224 patients were found to meet inclusion and exclusion criteria.

Data collected include age, sex, number of physical therapy visits, Orthopedic Certified Specialist (OCS) status of the evaluating physical therapist, and the presence or absence of six CPG-recommended interventions for mechanical neck pain (thoracic manipulation, cervical mobilization, TENS, dry needling, advice to stay active, and scapular resistance exercise). Advice to stay active was defined as any home exercise program (HEP) given to the patient. Each of the six CPG interventions was recorded as present if the intervention was performed at least once during the entire bout of physical therapy. Furthermore, other outcome data include pain before and after physical therapy, total cervical rotation active ROM before and after physical therapy, and Neck Disability Index (NDI) score (listed as a percentage) before and after physical therapy. Total cervical rotation ROM was created by adding up a both right and left rotation to help reduce errors in measuring rotation ROM. Because most post-therapy measurements are obtained in the final session of therapy-a session which was missing for many patients who abruptly discontinued therapy-we also performed a subgroup analysis on outcomes for pain, ROM, and disability as expressed by the NDI to help get a more complete picture.

Statistical analysis was performed using SPSS software version 29.0. Frequency counts were used to determining the usage of CPG interventions among the cohort. One-way ANOVA with Tukey posthoc testing was used to compare patients receiving two or less, three, or four or more CPG interventions. The t-test was used to compare the number of CPG interventions used in comparison to the OCS status of the treating physical therapist.

## Results

**Figure 1:**
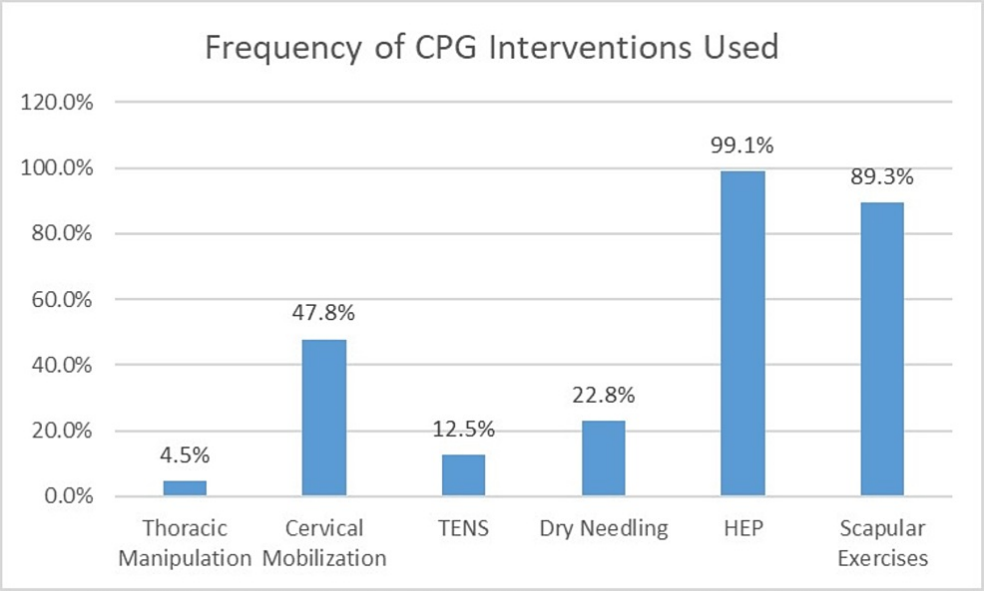
Graph of the frequencies of the six CPG interventions examined in the current study. Abbreviations: TENS - transcutaneous electrical nerve stimulation, HEP - home exercise program

Patients (n=224) in the current study had an average age (standard deviation, SD) of 60.7 years (16.2 years) with 71.0% of patients (n=159) being female and 29.0% of patients (n=65) being male. Patients had an average (SD) number of PT visits of 7.1 visits (4.2 visits). Of the entire cohort, 76 patients (n=33.9%) had chronic neck pain, which we defined as pain lasting in duration equal to or longer than 180 days. For treatment selection, 4.5% of patients (n=10) received thoracic manipulation, 47.8% of patients (n=107) received cervical mobilization, 12.5% of patients (n=28) received TENS, 22.8% of patients (n=51) received dry needling, 99.1% of patients (n=222) received a HEP with advice to stay active, and 89.3% of patients (n=200) received scapular resistance exercises (Table 1, Figure 1).

**Table 1:** Frequencies (values and percentages) of CPG interventions used among the cohort. Abbreviations: TENS - transcutaneous electrical nerve stimulation, HEP - home exercise program.

	Received (n)	Received (%)	Did Not Receive (n)	Did Not Receive (%)
Thoracic Manipulation	10	4.5%	214	95.5%
Cervical Mobilization	107	47.8%	117	52.2%
TENS	28	12.5%	196	87.5%
Dry Needling	51	22.8%	173	77.2%
HEP	222	99.1%	2	0.9%
Scapular Exercises	200	89.3%	24	10.7%

For number of interventions used per patient, 3.1% of patients (n=7) received one intervention, 37.9% of patients (n=85) received two interventions, 41.5% of patients (n=93) received three interventions, 14.7% of patients (n=33) received four interventions, 2.7% of patients (n=6) received five interventions, and none of the patients received all six interventions (Table 2).

**Table 2:** Frequency of number of CPG interventions used for the cohort during a bout of physical therapy.

	Frequency (n)	Frequency (%)
One CPG Intervention Received	7	3.1%
Two CPG Interventions Received	85	37.9%
Three CPG Interventions Received	93	41.5%
Four CPG Interventions Received	33	14.7%
Five CPG Interventions Received	6	2.7%
Six CPG Interventions Received	0	0.0%

 For subgroup analysis, three subgroups based on outcomes were created: Pain (n=192), range of motion (n=96), and Neck Disability Index disability score (n=77). There was no significant difference in improvement in pain based on the number of CPG interventions used during the bout of PT (p=0.56). However, one-way ANOVA testing revealed a significant difference in the amount of PT visits used for pain outcomes (p<0.01). Patients who received four or more CPG interventions had significantly more visits than patients who received two or less CPG interventions during the bout of PT (p<0.01). There was no significant difference in improvement in ROM based on the number of CPG interventions used during the bout of PT (p=0.74). Furthermore, there was no significant difference in the amount of PT visits used based on the number of CPG interventions used (p=0.21). Finally, there was no significant difference in the improvement in disability per the Neck Disability Index based on the number of CPG interventions used during the bout of PT (p=0.17). However, patients who received four or more CPG interventions had significantly more PT visits with the same outcomes as compared to patients using three or two or less CPG interventions (p=0.02 and p<0.01).

To further understand the different therapy utilization rates, patients were divided into groups depending on the OCS status of the treating physical therapist who did the evaluation. The OCS Group (n=16) and the Non-OCS group (n=174) had a significant difference in the amount of CPG interventions used (p<0.01), with the OCS group utilizing more CPG interventions (3.4 interventions compared to 2.7 interventions).

## Discussion

Neck pain is a disabling condition that has received much attention in the literature in an effort to improve patient management via the creation and revision of a Neck Pain CPG by the APTA [1,2]. The current study demonstrates that despite the significant effort to create recommended guidelines for the treatment of neck pain, CPG-recommended interventions are used with varying frequency. Some interventions, such as thoracic manipulation, are rarely used while other interventions, such as advice to stay active and scapular resistance exercises, are used for nearly every patient. Other studies in the literature have substantiated our findings, namely that CPGs are poorly utilized [6,8]. In fact, there are multiple studies aimed at improving adherence to CPG and removing barriers to CPG utilization [8,11,12]. One such strategy involves performing peer assessment to help improve awareness and reflection [8]. However, even if such measures were successful and adherence were to improve, our data found that those improvements would not have actually been clinically significant in terms of improving patient outcomes. Our data showed that the number of CPG interventions used during the bout of physical therapy did not correlate with statistically significant improvement in pain, cervical ROM, and overall function. These outcomes are supported by a study in the literature that found that the use of a CPG for neck and low back pain was not associated with final pain and disability scores, but use was associated with a mild increase in physical therapy visits [4].

Our data showed that as the number of therapy visits increased, so too did the number of CPG interventions used and that neither of those variables was associated with improvement in pain. There was also a significant association between the increased number of physical therapy visits and the increased number of CPG-recommended treatments utilized during the bout of physical therapy. Depending on the outlook, the increased visits could be a positive or a negative attribute. Increased visits could indicate greater attendance to physical therapy visits because of higher quality physical therapy care, or it could indicate greater waste if the outcomes are the same, despite the increase in visits. Interestingly, that study by Beneciuk et al. found that the overall CPG treatment adherence rate that was relatively high (71.2%), which is contrary to the current study [4].

There are several limitations of the current study. First, the study has a relatively small sample size from a single hospital system. Therefore, the results may not be generalizable to physical therapists across different regions. Furthermore, while the study included patients from multiple outpatient clinics, it is unknown how many physical therapists or physical therapist assistants participated in the treatment of the 224 patients in the study. Therefore, it is unknown if the problem is more widespread among physical therapists or just represents consistent poor adherence among a smaller number of physical therapists. It is also possible that some physical therapists are simply not aware of the current CPG. Strengths of the current study include the retrospective nature of the study compared to the surveys, which has been the usual way of assessing CPG adherence in the literature [6,9,10]. According to Maas et al., physical therapists have been shown to not hold realistic perceptions of their adherence to CPGs [8]. Also, the current study attempted to be as conservative as possible when assessing CPG treatment adherence as the authors’ hypothesis was that adherence to CPG-recommended treatments would be low. A treatment was counted as completed by the authors even if the treatment was only applied one time during the entire bout of physical therapy, which had an average of seven visits. Therefore, the current study does not comment on the quality and duration of the use of the CPG-recommended treatments, possibly indicating that the utilized CPG treatments may not have been used effectively. This fact would impact the overall outcomes and could explain why there was no significant difference in pain, ROM, or function based on the number of CPG used during the bout of physical therapy. If this assumption is true, the actual effective utilization of CPG-recommended treatments would be even lower than the current study proves. This may be why the literature shows that utilizing other CPGs, such as one for low back pain, leads to improved outcomes with lower costs [13]. More research is needed to determine how the duration and overall use of the CPG impact patient outcomes.

## Conclusions

CPG interventions are used with varying frequency in patients treated by physical therapists for neck pain with mobility deficits. Thoracic manipulation is rarely used whereas advice to stay active and scapular resistance exercises are a common part of PT treatment for mechanical neck pain. The number of CPG interventions used during the treatment session did not impact outcomes for pain, ROM, and function. Furthermore, increased CPG intervention usage was associated with increased patient visits, despite no significant improvement in outcomes. Patients treated by an evaluating PT who had their OCS were more likely to be treated with more CPG interventions. More research is needed to determine impact of CPG on outcomes as well as reasons for adherence or non-adherence to CPG interventions.
